# Emodin and Aloe-Emodin Reduce Cell Growth and Disrupt Metabolic Plasticity in Human Melanoma Cells

**DOI:** 10.3390/nu17071113

**Published:** 2025-03-22

**Authors:** Federica Baldassari, Marcella Bonanomi, Sara Mallia, Matteo Bonas, Elisa Brivio, Tecla Aramini, Danilo Porro, Daniela Gaglio

**Affiliations:** 1 Institute of Bioimaging and Complex Biological Systems, National Research Council (CNR), 20054 Segrate, MI, Italy; federica.baldassari@cnr.it (F.B.); marcella.bonanomi@cnr.it (M.B.); sara.mallia@cnr.it (S.M.); teclaaramini@cnr.it (T.A.); danilo.porro@unimib.it (D.P.); 2 National Biodiversity Future Center (NBFC), 90133 Palermo, PA, Italy; 3 Department of Biotechnology and Bioscience, University of Milano-Bicocca, 20126 Milano, MI, Italy; matteo.bonas@unimib.it (M.B.); elisa.brivio1@unimib.it (E.B.)

**Keywords:** melanoma, metabolic rewiring, metabolomics, phenotype shifting, treatment vulnerabilities, natural compounds, anticancer drugs, biodiversity

## Abstract

**Background/Objectives**: Melanoma is an aggressive skin cancer with intratumor metabolic heterogeneity, which drives its progression and therapy resistance. Natural anthraquinones, such as emodin and aloe-emodin, exhibit anti-cancer properties, but their effects on metabolic plasticity remain unclear. This study evaluated their impact on proliferation and metabolic pathways in heterogenous melanoma human cell lines. **Methods**: COLO 800, COLO 794, and A375 melanoma cell lines representing distinct metabolic phenotypes were analyzed. Targeted and untargeted metabolomics analyses integrated with Seahorse assays were performed to assess the effects of emodin and aloe-emodin on cell proliferation, mitochondrial function, and redox homeostasis. Glucose tracing using [U-^13^C_6_] glucose and metabolic flux analysis (MFA) were carried out to evaluate the glycolysis and TCA cycle dynamics. **Results**: Emodin and aloe-emodin inhibited proliferation by disrupting glycolysis, oxidative phosphorylation, and energy production across all cell lines. Both compounds impaired glucose metabolism, reduced TCA cycle intermediates, and induced mitochondrial ROS accumulation, causing oxidative stress and redox imbalance. Despite intrinsic metabolic differences, COLO 800 and COLO 794 upregulated antioxidant defenses; A375 enhanced one-carbon metabolism and amino acid pathways to maintain redox balance and nucleotide biosynthesis. **Conclusions**: Emodin and aloe-emodin can disrupt the metabolic plasticity of melanoma cells by impairing glycolysis, mitochondrial function, and redox homeostasis. Their ability to target metabolic vulnerabilities across diverse phenotypes highlights their therapeutic potential for overcoming resistance mechanisms and advancing melanoma treatment strategies.

## 1. Introduction

Melanoma is the most aggressive and deadliest form of human skin cancer, characterized by rapid growth and a high incidence rate [1]. Its aggressive nature is partially attributed to the ability of melanoma cells to adopt diverse phenotypes during tumor growth and metastasis [2]. These cells originate from the malignant transformation of melanocytes, driven by oncogenic signaling pathways and cancer-associated metabolic reprogramming, which are intricately linked processes. Among these pathways, MAPK signaling is frequently activated, primarily due to mutations in BRAF (~50%), NRAS (~20%), and NF1 (~10–15%) [3,4]. BRAF mutations are closely associated with enhanced glycolytic metabolism, driving melanoma cells to metabolize up to 80% of their glucose into lactate, even under normoxic conditions [5]. The metabolic heterogeneity of melanoma cells is also influenced by their division rate, leading to the classification of two distinct groups: fast-cycling cells reliant on high glycolytic activity and slow-cycling cells that depend on mitochondrial oxidative phosphorylation (OXPHOS) [6]. This metabolic plasticity contributes to the aggressiveness of melanoma and its resistance to conventional therapies, which often fail to achieve curative outcomes [6].

In recent years, natural products have gained significant attention for their therapeutic potential in cancer due to their low toxicity and multitargeted mechanisms of action [7]. Quinones, a class of redox-active organic compounds, are particularly interesting in cancer research due to their ability to participate in electron transfer reactions [8]. Among these, anthraquinones such as emodin and aloe-emodin, derived from plants like *Rheum palmatum* (rhubarb) and *Aloe vera*, have been extensively studied for their pharmacological properties [9]. Emodin and aloe-emodin can, through their quinone moiety, disrupt electron transport processes in cancer cells, thereby altering the cellular redox balance and promoting oxidative stress [8,9]. Furthermore, the hydroxyl groups of these molecules enable the formation of hydrogen and ionic bonds, facilitating interactions with a variety of cellular targets, including enzymes, transporters, ion channels, and receptors, interfering with cell proliferation and metabolism [10,11,12].

In this study, using three melanoma cell lines (COLO 800, derived from a primary tumor; COLO 794, derived from a metastasis from the same patient; and A375, derived from a nodule metastasis from a different patient), we investigated the anti-proliferative effects of emodin anthraquinones in the context of metabolic heterogeneity. The results demonstrate that emodin and aloe-emodin inhibit melanoma cell proliferation by targeting key metabolic pathways and altering redox homeostasis (see Graphical Abstract). These effects were independent of the intrinsic metabolic phenotypes of the cell lines, as both compounds consistently impaired glucose metabolism, mitochondrial function, and energy production. Moreover, this study highlights the differential metabolic responses of melanoma cells to treatment, including shifts into the amino acid metabolism, glutathione homeostasis, and one-carbon metabolic pathways, providing insights into their adaptive strategies under metabolic stress. Furthermore, the study underscores the therapeutic potential of emodin anthraquinones as metabolic modulators capable of overcoming the inherent metabolic flexibility of melanoma.

## 2. Materials and Methods

### 2.1. Cell Culture

The COLO 800 and COLO 794 cell lines were grown in Roswell Park Memorial Institute (RPMI) 1640, while the A375 cell line was grown in Dulbecco’s modified Eagle’s medium (DMEM). All media were supplemented with 2 mM L-glutamine, 10% fetal bovine serum (FBS), 100 U/mL penicillin, and 100 μg/mL streptomycin. The cells were grown at 37 °C in a 5% CO_2_ incubator. The cell lines were obtained from the American Type Culture Collection (ATCC) (LGC Chemicals Standard, Teddington, UK). The cell culture reagents were purchased from Life Technologies (Waltham, MA, USA).

### 2.2. Cell Proliferation Analysis

To obtain the proliferation curves under nutrient deprivation conditions, the cells were plated in 6-well plates. The medium was replaced after 24 h with either normal growth medium, low-glutamine medium (0.5 mM Gln), or low-glucose medium (1 mM Glc). The cells were collected and counted after 24, 48, 72, and 144 h. To obtain the dose–response curves, the cells were exposed to either aloe-emodin (Aloe-Emo) at concentrations of 0, 5, 10, 15, 20 μM or emodin (F-Emo) at 0, 10, 20, 30, 40, and 50 μM for 48 h. To obtain the long-term proliferation curves in the presence of the compounds, the cells were plated in 12-well plates and, after 24 h, Aloe-Emo or F-Emo was added at the indicated concentrations. The cells were collected and counted after 24, 48, 72, and 144 h. The emodin and aloe-emodin were purchased from Sigma-Aldrich (St. Louis, MO, USA).

### 2.3. Oxygen Consumption Rate Analysis

The cellular oxygen consumption rate (OCR) was measured using a Seahorse XF extracellular flux analyzer (Seahorse Bioscience Inc., North Billerica, MA, USA) according to the manufacturer’s instructions. Briefly, cells were seeded in Seahorse XF 24-well assay plates and treated according to the experiment. On the day of the assay, the medium was washed and replaced with a pre-warmed assay medium (non-buffered DMEM supplemented with 1 mM sodium pyruvate, 25 mM glucose, and 2 mM glutamine, pH 7.4), and incubated in a non-CO_2_ incubator at 37 °C for 60 min. The mitochondrial stress was assessed using the Seahorse XF Cell Mito Stress Test Kit which includes oligomycin (1 μM), an inhibitor of ATP synthase; FCCP (1 μM), an uncoupler; and rotenone/antimycin A (0.5 μM), which are electron transport inhibitors. The following formulas were used to calculate respiratory parameters: Basal Respiration = OCR_basal_-OCR_rot/ant_; Maximal Respiration = OCR_FCCP_-OCR _rot/ant_; Spare Respiratory Capacity = Maximal respiration-Basal Respiration; ATP Mitochondrial Production: OCR_basal_-OCR_oligo_. In these formulas, OCR_basal_ refers to the OCR recorded before any drugs were added; OCR_oligo_ refers to the OCR recorded after the oligomycin injection; OCR_FCCP_ refers to the OCR recorded after the FCCP injection; and OCR_rot/ant_ refers to the OCR recorded after the rotenone/antimycin injection.

### 2.4. ROS Level Measurements

The total ROS levels and mitochondrial ROS levels were measured using the Dichloro-dihydro-fluoresceine-diacetate (DCFDA) Cellular ROS Detection Assay Kit (Abcam, Cambridge, UK) and MitoSOX™ Red Mitochondrial Superoxide Indicator (Thermo Fisher Scientific, Waltham, MA USA), respectively. The cells were stained with 20 μM DCFDA or 5 μM MitoSOX™ Red for 30 min at 37 °C. Then, the cells were washed and collected in PBS/5%FBS for analysis. Ten thousand gated events were analyzed by flow cytometry on a CytoFlex S (Beckman Coulter, Brea, CA, USA), using the FITC channel for DCFDA and PE channel for MitoSOX™ Red. The median fluorescence intensity of the cells was determined using CytExpert 2.0 software (Beckman Coulter).

### 2.5. Autophagy

The autophagy levels were assessed using the CYTO-ID^®^ Autophagy Detection Kit (Abcam). The cells were washed and the medium was replaced with the assay buffer. The samples were stained with a CYTO-ID^®^ probe (1:1000) and placed at 37 °C for 30 min. The cells were collected, washed, and fixed in a 10% formaldehyde solution for 20 min at room temperature. The samples were washed again and collected in the assay buffer for analysis. Ten thousand gated events were analyzed by flow cytometry on a CytoFlex S (Beckman Coulter, Brea, CA, USA) using the FITC channel. The median fluorescence intensity of the cells was determined using CytExpert 2.0 software (Beckman Coulter, Lane Cove West, NSW, Australia).

### 2.6. Metabolites Quantification in the Media Samples

The absolute glucose, lactate, glutamine, and glutamate levels in the spent media were determined enzymatically using the YSI2950 bioanalyzer (YSI Incorporated, Yellow Springs, OH, USA). Media collected from the experiments were thawed and centrifuged at 2000× *g* for 5 min before the analysis. The YSI bioanalyzer employs enzyme-based biosensors to measure the glucose, lactate, glutamate, and glutamine concentrations. The biosensors use oxidase-containing membranes for oxidizing substrates, releasing hydrogen peroxide. The hydrogen peroxide is detected amperometrically on a platinum electrode surface. The current flow at the electrode is directly proportional to the hydrogen peroxide concentration and hence to the substrate concentration. Glucose, lactate, glutamine, and glutamate standard solutions were used to calibrate the instrument. Glucose and glutamine consumption, as well as lactate and glutamate release, were calculated as follows: consumption = mmol/L of compound in fresh complete media—mmol/L of compound in cultured media; release = mmol/L of compound in cultured media—mmol/L of compound in fresh complete media. The rates were reported as mmol/L per 10^6^ cells.

### 2.7. Metabolite Extraction from Cell Culture

For the untargeted experiments, the cells were plated in 6-well plates with normal growth medium, which was replaced after 24 h with complete fresh medium in the presence or the absence of the treatments, and then incubated for 48 h. For the labeling experiments, the cells were incubated for 48 h in fresh media supplemented with 25 mM [U-^13^C_6_]glucose or 2 mM [U-^13^C_5_]glutamine (purchased from Cambridge Isotope Laboratories, Tewksbury, MA, USA) in the presence or the absence of the treatments. For metabolite extraction for LC-MS analysis, the cells were quickly rinsed with 0.9% NaCl and quenched with 500 μL of ice-cold 70:30 acetonitrile/water. The plates were placed at −80 °C for 10 min, collected by scraping, sonicated twice for 5 s with five pulses at 70% power, and then centrifuged at 12,000× *g* for 10 min at 4 °C. The supernatant was collected in a glass insert and dried in a centrifugal vacuum concentrator (Concentrator plus/Vacufuge plus, Eppendorf, Hamburg, Germany) at 30 °C for about 2.5 h. The samples were then resuspended with 150 μL of H_2_O before the analyses.

### 2.8. LC-MS Metabolic Profiling

The dried samples were resuspended with 150 µL of H_2_O and then analyzed using a UHPLC-QTOF mass spectrometer. LC separation was performed using an Agilent 1290 Infinity UHPLC system and an InfintyLab Poroshell 120 PFP column (2.1 × 100 mm, 2.7 μm; Agilent Technologies, Santa Clara, CA, USA). Mobile phase A was water with 0.1% formic acid. Mobile phase B was acetonitrile with 0.1% formic acid. The injection volume was 15 μL and the LC gradient conditions were 0 min: 100% A; 2 min: 100% A; 4 min: 99% A; 10 min: 98% A; 11 min: 70% A; 15 min: 70% A; and 16 min: 100% A with 5 min of post-run. The flow rate was 0.2 mL/min and the column temperature was 35 °C. MS detection was performed using an Agilent 6550 iFunnel Q-TOF mass spectrometer with a Dual JetStream source operating in the negative ionization mode. The MS parameters were gas temp: 285 °C; gas flow: 14 L/min; nebulizer pressure: 45 psig; sheath gas temp: 330 °C; sheath gas flow: 12 L/min; VCap: 3700 V; Fragmentor: 175 V; Skimmer: 65 V; and Octopole RF: 750 V. Active reference mass correction was performed through a second nebulizer using masses with *m*/*z* values of 112.9855 and 1033.9881. Data were acquired from *m*/*z* 60 to 1050. The data analysis and isotopic natural abundance correction were performed using MassHunter ProFinder and MassHunter VistaFlux software (version 10.0.2) (Agilent Technologies), as described in [13].

### 2.9. Metabolomics Statistical Data Analysis

The metabolomics data were analyzed using Metaboanalyst 5.0 [13] (https://www.metaboanalyst.ca/, accessed on 24 May 2024) and Mass Profiler Professional 15.1 software (Agilent Technologies). The raw data were transformed into the log2 scale and normalized using Pareto scaling. The data were then filtered, and the entities that were present in at least 80.0 percent of the samples in one condition were retained for the analysis. The statistical analysis was performed by applying an unpaired *t*-test or one-way ANOVA with a *p*-value cut-off of 0.05. Data visualization of the significant entities was performed using a hierarchical clustering algorithm. The list of significant entities was subjected to enrichment analysis using MetaboAnalyst. Over Representation Analysis (ORA) was performed using a metabolite set library derived from SMPDB (The Small Molecule Pathway Database), which included 99 metabolite sets based on normal human metabolic pathways. The ORA was implemented using the hypergeometric test to evaluate whether a particular metabolite set was represented more than expected by chance within the given compound list. One-tailed *p* values were provided after adjusting for multiple tests.

### 2.10. ^13^C Metabolic Flux Analysis

We used ^13^C-labeled substrates to trace how carbon flows through the cell’s key metabolic pathways, specifically glycolysis, the TCA cycle, and the reactions involved in building cell biomass. ^13^C MFA was performed for a metabolic network consisting of the glycolysis, TCA, and biomass synthesis pathways [14] according to the Elementary Metabolite Unit (EMU) framework [15]. This approach helped us understand and calculate the complexity of mammalian carbon flow in cells. Metabolic fluxes were estimated by the least-squares regression of the metabolite labeling and extracellular flux data. The flux values were iteratively adjusted using a Levenberg–Marquardt algorithm to minimize the sum of the squared residual objective function. The best global fit was found after estimating the best local fit at least 100 times using random initial values for all reactions in the network. All the fluxes were subjected to the chi-square statistical test to assess the goodness of fit, and 95% confidence intervals were computed [16]. The computation was carried out using INCA 2.3 [17] software. Supplementary Tables S1 and S2 provide the quantitative flux values (expressed in pmol·h^−1^·10^4^ cells) of the reaction in the network model and confidence intervals for each estimated flux. The experiments were conducted under steady-state conditions, meaning that the levels of intracellular metabolites and fluxes remained constant over time. This assumption ensures that the ^13^C labeling reached equilibrium, making our flux estimates more reliable.

### 2.11. Lactate Dehydrogenase and Pyruvate Dehydrogenase Quantification

Using the manufacturer’s protocol, the total cellular LDH and PDH levels were measured using the Lactate Dehydrogenase Activity Colorimetric Assay Kit and Pyruvate Dehydrogenase Activity Colorimetric Assay, respectively (BioVision Inc, Milpitas, CA, USA). Briefly, 500,000 cells were extracted by homogenization in 500 μL of assay buffer for LDH, and 100 μL for PDH. Volumes of 10 μL and 100 μL, respectively, were used for the assay. Colorimetric measurements were performed at OD 450 nm every 5 min up to 2 h using a Cary 60 ultraviolet–visible spectrophotometer (Agilent Technologies, Santa Clara, CA, USA).

### 2.12. Western Blot Analysis

Protein extraction was performed in RIPA buffer supplemented with a protease inhibitor cocktail. The proteins were separated on a 12% polyacrylamide gel and transferred to a nitrocellulose membrane. After blocking with 5% BSA in TBS-Tween, the membranes were incubated with the following antibodies: anti-AMPK-alpha (1:1000), anti-P-AMPKalpha (T172) (1:1000), and anti-β-actin (1:500) (Cell Signaling Technology, Danvers, MA, USA) overnight at 4 °C. Anti-rabbit fluorescent secondary antibody (1:15,000) (IRDye^®^ 800CW Donkey anti-Rabbit IgG, Li-Cor Biosciences, Lincoln, NE, USA) was used. The membranes were imaged using a Li-Cor Odyssey Fc scanner and densitometry analysis was performed using Image Studio Lite software, Version 5.2.

### 2.13. Statistical Data Analysis

Except for the analyses described in Section 2.9, all the other experiments were performed at least in triplicate. The data are presented as the mean ± standard deviation. The observed differences were tested for significance using the Student’s *t*-test (* *p* ≤ 0.01, ** *p* ≤ 0.005). The statistics are included in the figure legends.

## 3. Results

### 3.1. Melanoma Cancer Cell Lines: A Model of Cancer Metabolic Heterogeneity

**Figure 1:**
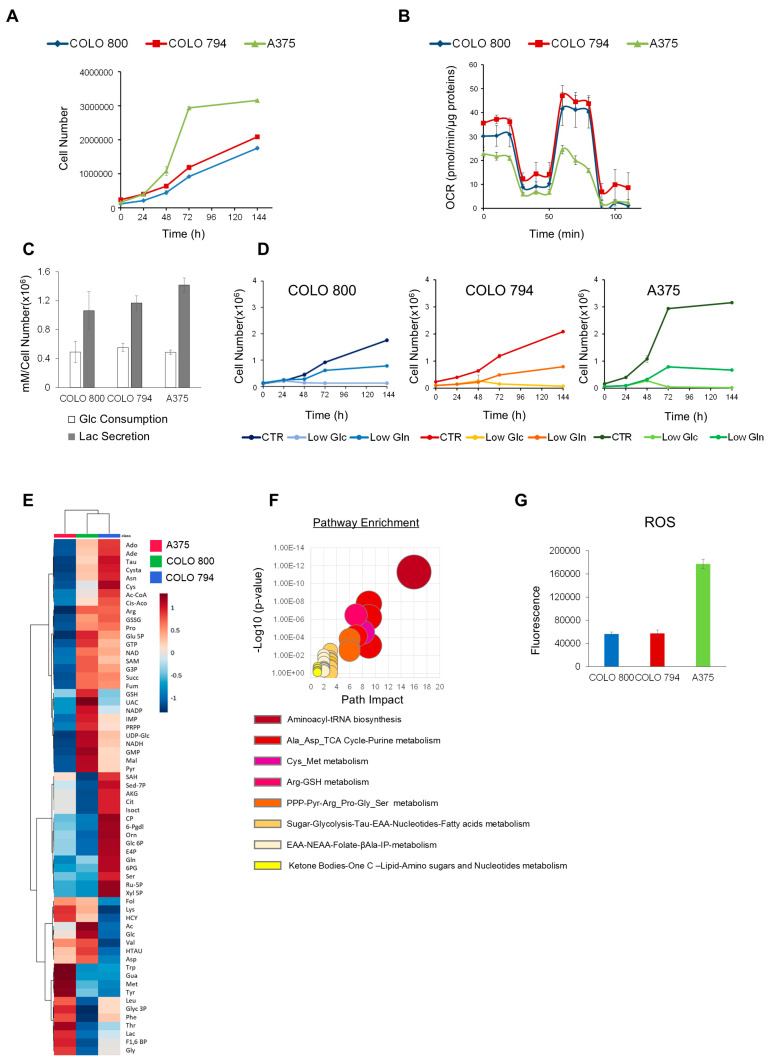
Metabolic phenotype characterization of melanoma cell lines. (**A**) Proliferation curves of COLO 800 (**―**), COLO 794 (**―**), and A375 (**―**) cell lines. The cells were grown in 6-well plates in the appropriate growth medium. The cells were collected and counted at the indicated time points. (**B**) Mitochondrial respiration reflected by OCR levels assessed by Mitostress Seahorse analysis under basal conditions in COLO 800 (**―**), COLO 794 (**―**), and A375 (**―**) cell lines. (**C**) Extracellular glucose uptake and lactate secretion in COLO 800, COLO 794, and A375 cells grown for 48 h, determined enzymatically using a YSI2950 bioanalyzer. (**D**) Proliferation curves of COLO 800, COLO 794, and A375 cells under normal, low-glucose (1 mM), and low-glutamine (0.5 mM) conditions. The cells were collected and counted at the indicated time points. (**E**) Hierarchical clustering heatmaps derived from untargeted metabolic profiling in COLO 800, COLO 794, and A375 cell lines grown in standard growth conditions. (**F**) Pathway enrichment from over-representation analysis of statistically different metabolites obtained from one-way ANOVA between COLO 800, COLO 794, and A375. (**G**) Intracellular ROS levels in COLO 800 (**―**), COLO 794 (**―**), and A375 (**―**) cells were measured by DCFDA staining. All data in the figure are expressed as the mean ± SD.

To investigate the different types of tumor metabolic rewiring in melanoma, we analyzed three human melanoma cell lines: COLO 800 (derived from a primary subcutaneous nodule), COLO 794 (derived from a subcutaneous metastasis from the same individual as COLO 800), and A375 (derived from a metastatic nodule from a different individual) (Figure S1A). Consistent with their shared origin, COLO 800 and COLO 794 exhibited similar proliferation rates and metabolic behaviors in contrast to A375 (Figure 1A,B). The A375 melanoma cell line showed a higher proliferation rate, consistent with the “Warburg effect” paradigm characterized by reduced oxidative phosphorylation and increased conversion of glucose to lactate (Figure 1B,C). Despite all three cell lines displaying comparable glucose dependency (Figure 1D), the untargeted metabolic profiling revealed different glucose utilization rates (Figure 1E). The metabolic signature, in fact, highlighted key differences between the cell lines, with COLO 800 and COLO 794 showing higher levels of TCA cycle intermediates, consistent with enhanced mitochondrial respiration (Figure 1E). This contrasts sharply with the A375 cells, which showed reduced levels of these metabolites alongside an increase in glycolytic intermediates and folate cycle metabolites (Figure 1E). Moreover, the increased levels of amino acids, such as arginine, proline, cysteine, asparagine, lysine, and methionine, in COLO 800 and COLO 794 compared with A375 further emphasize their reliance on distinct metabolic programs to maintain anabolic processes (Figure 1E). Despite their shared origin, COLO 800 and COLO 794 exhibited different antioxidant defenses. Specifically, the elevated levels of pentose phosphate pathway (PPP) intermediates in COLO 800 suggest a greater reliance on this pathway for managing oxidative stress (Figure 1E). On the other hand, the A375 cells exhibited an enrichment of ketone bodies, a well-known metabolic adaptation to cellular stress or nutrient deprivation (Figure 1F). Furthermore, the elevated basal ROS levels (Figure 1G) and reduction in redox scavenger metabolites observed in the A375 cells may contribute to their enhanced proliferative capacity (Figure 1A).

### 3.2. Emodin and Aloe-Emodin Inhibit Energetic Metabolic Pathways Essential for Melanoma Cell Proliferation

**Figure 2:**
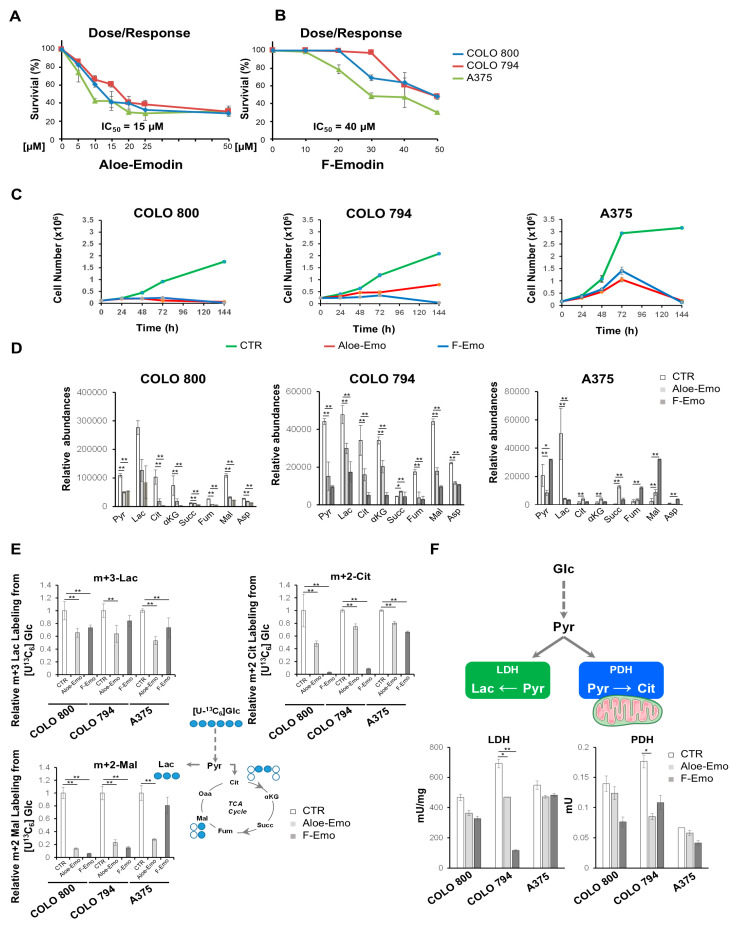
Metabolic characterization of melanoma cell lines after aloe-emodin (Aloe-Emo) or emodin (F-Emo) treatment. (**A,B**) Dose–response curves of COLO 800, COLO 794, and A375 cell lines after Aloe-Emo (**A**) or F-Emo (**B**) treatment for 48 h at the indicated concentrations. (**C**) Proliferation curves of COLO 800, COLO 794, and A375 cell lines under normal conditions (**―**) or in the presence of 15 μM Aloe-Emo (**―**) or 40 μM F-Emo (**―**). The cells were collected and counted at the indicated time points. (**D**) Relative abundances of pyruvate, lactate, TCA intermediates, and aspartate obtained by LC-MS in all three melanoma cell lines under normal conditions or after 40 μM F-Emo or 15 μM Aloe-Emo exposure. (**E**) Schematic representations and atomic transition map of relative isotope enrichment of metabolites from [U-^13^C_6_]glucose MFA (blue circles) in COLO 800, COLO 794, and A375 cell lines under normal conditions or in the presence of 15 μM Aloe-Emo or 40 μM F-Emo from LC-MS analysis. (**F**) Lactate dehydrogenase (LDH) and pyruvate dehydrogenase (PDH) activities obtained from colorimetric assay. All data in the figure are expressed as the mean ± SD. * *p* ≤ 0.01, ** *p* ≤ 0.005.

The natural anthraquinone derivatives emodin (1,3,8-trihydroxy-6-methylanthraquinone) (F-Emo) and aloe-emodin (1,8-dihydroxy-3-hydroxyl-methylanthraquinone) (Aloe-Emo) are the primary bioactive components found in *Rhamnus frangula* L. and *Aloe vera*, respectively [7,8,9]. These compounds share a characteristic tricyclic quinone structure and hydroxyl groups attached to the aromatic rings (Figure S1B), endowing them with unique bioactive properties. To study the potential effectiveness of bioactive compounds, specifically in cancer metabolic rewiring inhibition, we obtained dose–response curves for our melanoma cancer cell lines. All three melanoma cell lines (COLO 800, COLO 794, and A375) exhibited a dose-dependent growth arrest in response to increasing concentrations of F-Emo and Aloe-Emo (Figure 2A,B). For both compounds, there were no obvious differences in the IC50 values between the different cell lines. However, a comparison of the two compounds revealed that Aloe-Emo exhibited superior efficacy at lower concentrations, with an IC50 of approximately 15 μM, while F-Emo required a higher concentration to achieve the same effect, with an IC50 of 40 μM (see Figure 2A,B). Consistent with the literature data, Aloe-Emo induced autophagy in all the melanoma cell lines (Figure S1C) [18]. Despite differences in short-term effectiveness, the inhibitory effects of both compounds on melanoma cell growth converged over extended treatment periods. At 144 h in the long-term proliferation assays, both F-Emo and Aloe-Emo demonstrated similar growth-inhibiting effects across all three melanoma cell lines (Figure 2C). These results show that while Aloe-Emo exhibited stronger effects at lower doses during the initial stages of treatment, both compounds ultimately achieved comparable long-term inhibition of melanoma cell proliferation (Figure 2C). The metabolic profiles of the untargeted cell lines under the different treatments were consistent with their basic metabolic characterization and cellular origins. The analysis revealed a primary clustering of COLO 800 and COLO 794, which were distinctly separate from A375. Within this primary cluster, COLO 800 and COLO 794 formed distinct subclusters, further supporting the conclusion that, despite their shared origin, these cell lines exhibit subtle yet significant metabolic differences (Figure S1D).

**Figure 3:**
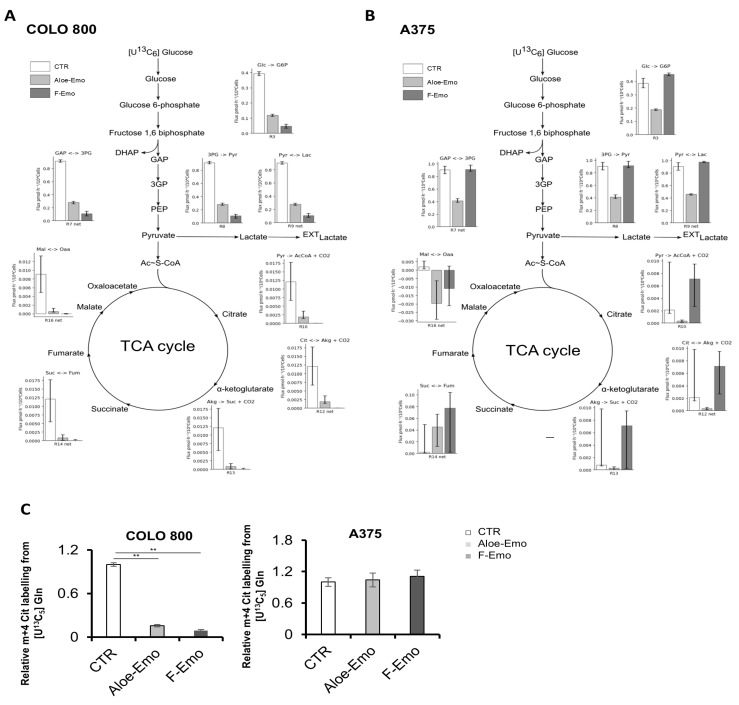
Estimated metabolic fluxes in central carbon metabolism for COLO 800 and A375. (**A**,**B**) Values of intracellular fluxes estimated by Metabolic Flux Analysis (MFA) using [U-^13^C_6_]glucose as tracer in COLO 800 (**A**) and A375 (**B**) cell lines. Bars indicate the 95% C.I. (**C**) Relative enrichment of m+4 citrate in COLO 800 and A375 using [U-^13^C_5_]glutamine. Data are expressed as the mean ± SD., ** *p* ≤ 0.005.

To further explore the effects of F-Emo and Aloe-Emo on metabolic rewiring, we performed a comprehensive metabolomics analysis. Notably, both treatments show significantly decreased levels of the metabolites from energetic pathways. Specifically, lactate was significantly reduced in all the cell lines, while intermediates of the TCA cycle were significantly reduced in COLO 800 and COLO 794 under both treatments compared to the control (Figure 2D). Using a [U-^13^C_6_]glucose tracer, we confirmed that both treatments effectively inhibited glucose metabolism by reducing glucose oxidation to lactate (m+3 lactate) in both cell lines. Furthermore, the reduced glucose-derived contributions to the TCA cycle, as evidenced by decreased levels of m+2 citrate and m+2 malate, suggest impaired TCA cycle activity and incomplete glucose oxidation (Figure 2E). This metabolic inhibition was further corroborated by the enzymatic activity assays, which showed a significant reduction in lactate dehydrogenase (LDH) and pyruvate dehydrogenase (PDH) activities in all three melanoma cell lines when treated with the two compounds (Figure 2F). Furthermore, the metabolic flux analysis (MFA) using [U-^13^C_6_]glucose confirmed a decreased glucose flux through lactate and reduced TCA flux in COLO 800 under both treatments, consistent with the observed metabolic rewiring (Figure 3A and Supplementary Table S1). On the other hand, the MFA performed using [U-^13^C_6_]glucose in the A375 cells revealed a distinct metabolic adaptation (Figure 3B and Supplementary Table S2). While glucose flux through lactate was significantly reduced with the Aloe-Emo treatment, the TCA cycle flux remained unchanged, probably due to compensatory glutamine metabolism, as shown by the m+4 citrate labeling using [U-^13^C_5_]glutamine (Figure 3C). The MFA was not conducted in the COLO 794 cells due to its similar metabolic behavior to COLO 800, as previously demonstrated in the metabolomic and Seahorse analyses.

Together, these results show that F-Emo and Aloe-Emo affect key energetic pathways, altering glucose metabolism and influencing the availability of metabolic intermediates. Moreover, they indicate differences in metabolic dependencies and responses among the melanoma cell lines following treatment.

### 3.3. Emodin and Aloe-Emodin Inhibit Mitochondrial Activity and Alter the Cellular Redox Balance

**Figure 4:**
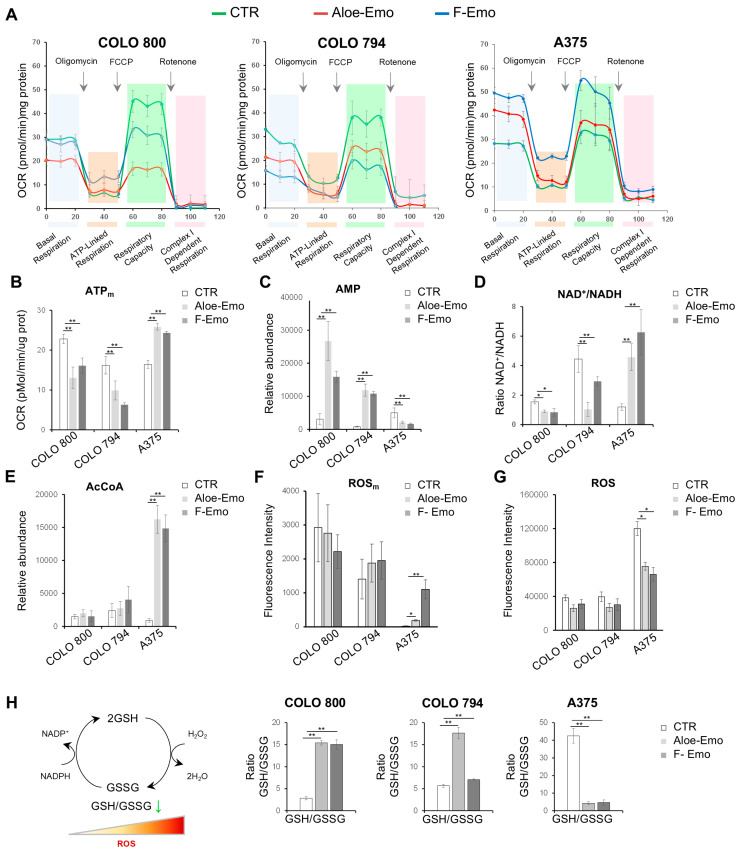
Respiratory and redox responses to emodin and aloe-emodin treatments. (**A**) Mitochondrial respiration reflected by OCR levels, assessed by Mitostress Seahorse analysis, under basal conditions or following the addition of oligomycin (1 μM), the uncoupler FCCP (1 μM), or the electron transport inhibitor rotenone (0.5 μM). The analysis was conducted on control (―) or treated (― and ―) melanoma cells. (**B**) ATP mitochondrial production reflected by OCR levels, as assessed by Mitostress Seahorse analysis, in all three melanoma cell lines under normal conditions or after exposure to the compounds. (**C**–**E**) Relative AMP abundance (**C**), NAD^+^/NADH ratio of NAD and NADH (**D**), and relative acetyl-CoA (AcCoA) abundance (**E**) obtained by LC-MS in all three melanoma cell lines under normal conditions or after F-Emo or Aloe-Emo exposure. (**F**,**G**) Mitochondrial ROS levels measured by MitoSOX™ Red staining (**F**) and total ROS levels measured by DCFDA staining (**G**) in COLO 800, COLO 794, and A375 cells under normal conditions or after Aloe-Emo or F-Emo exposure. (**H**) Schematic representation and relative GSH/GSSG ratio in COLO 800, COLO 794, and A375 cell lines under normal growth conditions or in the presence of Aloe-Emo or F-Emo obtained from LC-MS analysis. All data in the figure are expressed as the mean ± SD. * *p* ≤ 0.01, ** *p* ≤ 0.005.

To further evaluate the impact of F-Emo and Aloe-Emo on energy metabolism and redox homeostasis, a Seahorse analysis was conducted on all the melanoma cell lines (Figure 4A). COLO 800 and COLO 794, derived from the same patient, exhibited more similar mitochondrial behaviors under both treatments compared to A375, which was derived from a different individual.

The analysis of basal respiration, reflecting mitochondrial activity under normal conditions, showed a significant reduction in COLO 800 and COLO 794 following the treatments. This was accompanied by (i) a marked decrease in ATP-linked respiration, measured as the oligomycin-sensitive portion of oxygen consumption, indicating impaired ATP production via oxidative phosphorylation; (ii) a significant reduction in the maximal respiratory capacity, assessed by uncoupling the mitochondrial membrane potential with FCCP, indicating a reduction in the mitochondrial reserve capacity (Figure 4A). This disruption of mitochondrial activity was associated with decreased mitochondrial ATP levels and a significant accumulation of AMP (Figure 4B,C). Elevated AMP levels are consistent with the activation of AMP-activated protein kinase (AMPK), a key energy sensor, which may contribute to the anti-proliferative effects of F-Emo and Aloe-Emo in COLO 800 and COLO 794 (Figure S1E). In contrast, A375 exhibited distinct energy dynamics under both treatments: increased mitochondrial activity, accompanied by elevated mitochondrial ATP levels (Figure 4A,B); an increased NAD^+^/NADH ratio (Figure 4D), consistent with enhanced electron transfer from NADH to the electron transport chain and regeneration of NAD^+^ from NADH; and an increased abundance of acetyl-CoA (AcCoA) (Figure 4E). These findings show a metabolic response in A375 that is distinct from that of COLO 800 and COLO 794. In line with these findings, it is interesting to note that the A375 cell line exhibited increased mitochondrial ROS levels under both treatments, in contrast to the decreased total ROS levels (Figure 4F,G). To further investigate redox homeostasis, the glutathione levels were analyzed. While COLO 800 and COLO 794 displayed increased GSH/GSSG ratios, suggesting an ability to maintain physiological glutathione metabolism, A375 exhibited a complete inversion of physiological glutathione homeostasis (Figure 4H).

Taken together, these results show distinct metabolic and redox responses to emodin and aloe-emodin on our melanoma cell lines, with variations in mitochondrial behavior and energy homeostasis. We observed disruptions in oxidative phosphorylation, ATP dynamics, and the ROS balance after treatment.

### 3.4. Metabolic Pathway Activation to Support Redox Homeostasis

**Figure 5:**
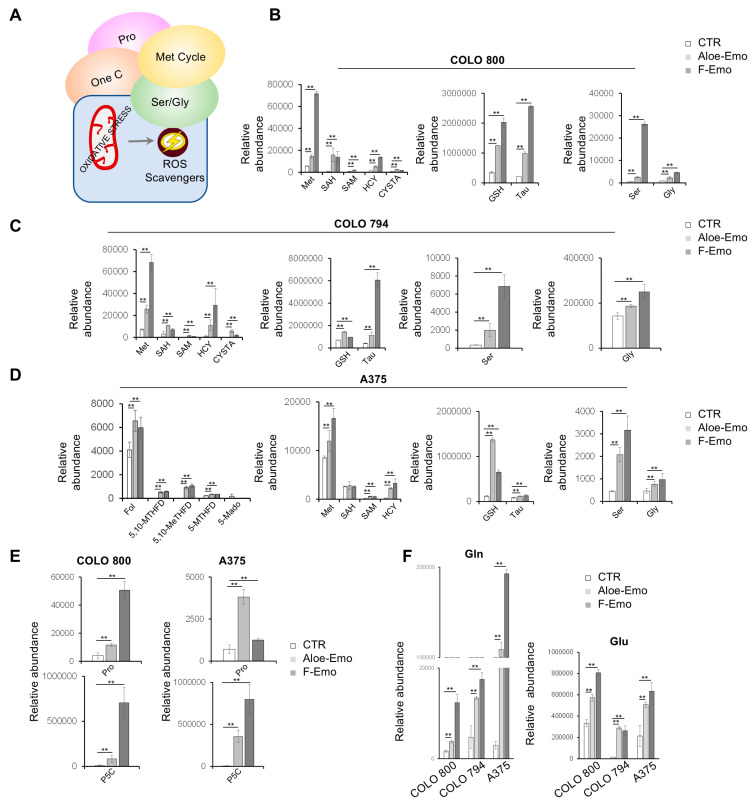
Metabolic pathway activation to support redox homeostasis for cell survival. (**A**) Schematic representation of ROS-scavenging pathways involved in redox homeostasis. (**B**–**D)** Relative abundances of metabolites involved in one-carbon metabolism, the methionine cycle, redox metabolism, and serine–glycine metabolism in COLO 800 (**B**), COLO 794 (**C**), and A375 (**D**) obtained from LC-MS analysis under normal conditions or in the presence of Aloe-Emo or F-Emo. (**E**) Relative abundances of proline and pyrroline-5-carboxylic acid in COLO 800 and A375 obtained from LC-MS analysis under normal conditions or in the presence of Aloe-Emo or F-Emo. (**F**) Relative abundance of glutamine and glutamate obtained from LC-MS analysis under normal conditions or in the presence of Aloe-Emo or F-Emo. All data in the figure are expressed as the mean ± SD, ** *p* ≤ 0.005.

The results obtained so far indicate changes in metabolic pathways related to redox homeostasis, prompting further investigation. The targeted metabolomics analysis revealed significant upregulation of key metabolites associated with the methionine cycle, as well as serine, glycine, taurine, and proline metabolism after the treatments (Figure 5A–E). Elevated levels of the amino acids glutamine and glutamate were also detected, implying an activation of cancer-related rewiring of glutamine metabolism (Figure 5F). In addition, both COLO 800 and COLO 794 showed increased levels of amino acids, such as tyrosine, lysine, phenylalanine, leucine, serine, and methionine, as well as the redox-related metabolites hypotaurine and reduced riboflavin, suggesting a shared response to the treatments in terms of amino acid metabolism and redox regulation (Figure S2A,B).

Interestingly, the A375 cells exhibited a broader metabolic response, not only encompassing the pathways mentioned previously but also an upregulation of folate metabolism (Figure 5D). Furthermore, the A375 cells exhibited significantly increased levels of arginine, serine, proline, asparagine, and threonine (which play crucial roles in biosynthetic processes, redox balance, and energy production) under the emodin and aloe-emodin treatments (Figure S2C). These changes highlight more pronounced and unique metabolic reprogramming in A375 compared to COLO 800 and COLO 794, reflecting the ability of A375 cells to adapt their metabolism under treatment-induced stress (Figure S2C).

Together, these findings indicate metabolic changes in our melanoma cell lines in response to mitochondrial ROS accumulation, with increased activity in the amino acid and methionine cycle-related pathways.

## 4. Discussion

This study leveraged a systems metabolomics approach to provide novel insights into the metabolic heterogeneity of melanoma cells and the therapeutic potential of anthraquinones, specifically emodin and aloe-emodin, as modulators of metabolic rewiring and redox homeostasis. The bioactive compound emodin that is found in plants, such as *Rheum palmatum (rhubarb)* and *Aloe vera*, has been extensively studied for its pharmacological properties, including anti-inflammatory, antimicrobial, and antitumor effects [19]. In this study, we determined how melanoma cells respond metabolically to treatment with emodin from *Frangula* (F-Emo) and aloe-emodin from *Aloe vera* (Aloe-Emo), which was affected by their intrinsic heterogeneity. In fact, the quinone structure of emodin and aloe-emodin facilitates redox cycling and ROS generation, providing a mechanistic basis for selectively targeting cancer cells, which are often characterized by elevated oxidative stress and aberrant metabolic adaptations [20].

Three melanoma cell lines, COLO 800, COLO 794, and A375, were used to reveal the pronounced metabolic heterogeneity that underlies the adaptability of melanoma cells to different microenvironmental and intrinsic stressors. COLO 800 and COLO 794, derived from the same patient but differing in tumor stage and location, displayed metabolic profiles dominated by oxidative phosphorylation (OXPHOS) and mitochondrial respiration. Some of the distinct metabolic behaviors observed between COLO 800 and COLO 794 could be attributed to their “primary versus metastatic” origin, where COLO 800, as a primary tumor-derived cell line, may exhibit enhanced PPP activity to prepare for the oxidative stress that is commonly encountered during tumor initiation. In contrast, COLO 794, derived from a metastasis, may have adopted antioxidant strategies that are less dependent on the PPP, potentially reflecting its metabolic flexibility acquired during metastatic progression. In summary, COLO 800 was characterized by basic metabolic rewiring, while COLO 794 exhibited partial rewiring, occupying a transitional metabolic state, reflecting a dynamic balance between glycolysis and oxidative phosphorylation that may contribute to its metabolic adaptability and ability to metastasize.

In contrast, the A375 cells exhibited a glycolytic phenotype characterized by high lactate production, reduced mitochondrial respiration, and increased pentose phosphate pathway metabolites, aligning with the Warburg effect, a well-established metabolic hallmark of cancer [21] (Figure 1). Moreover, an increase in ketone bodies suggested the activation of an alternative energy pathway to glycolysis, reflecting the ability of the A375 cells to utilize this pathway as an energy source to sustain their metabolic demands under stress conditions. In the A375 cell line, a combination of oxidative stress and diminished antioxidant defenses was also observed. This behavior may paradoxically drive A375 cell proliferation by activating pro-survival signaling pathways and promoting metabolic plasticity.

The differential reliance on mitochondrial metabolism versus glycolysis underscores the different metabolic rewiring strategies of melanoma cells, which have not been well characterized in such detail in the context of an anthraquinone-based treatment. These findings extend our understanding of the interplay between tumor microenvironmental pressures and the metabolic plasticity of melanoma, which has been recognized as a key driver of therapeutic resistance [5,9].

Treatment with F-Emo and Aloe-Emo profoundly disrupted energy metabolism and redox homeostasis across all three cell lines but with notable differences in the responses. The compounds exhibited different inhibitory capacities, with Aloe-emo showing efficacy at lower doses. The inhibitory effect of Aloe-Emo at lower concentrations could be due to structural differences between the two anthraquinones, which influence their biological activity. Both compounds impaired glycolysis and OXPHOS, as evidenced by the reduced levels of TCA cycle intermediates, glycolytic end-products, and mitochondrial respiration (Figure 2 and Figure 4). These results are consistent with previous studies showing that emodin can directly induce mitochondrial dysfunction and inhibit components of the electron transport chain, including complex I [22,23]. This dual targeting of glycolysis and mitochondrial respiration is particularly interesting, as it exploits the metabolic plasticity of melanoma cells, leaving them vulnerable to energy crises and oxidative stress. A striking observation was the differential response of the cell lines to treatment-induced metabolic stress. COLO 800 and COLO 794 showed AMP accumulation including activation of AMPK signaling, which is indicative of a severe energy deficit (Figure 4 and Figure S1) [24]. Despite this, they maintained elevated GSH/GSSG ratios, highlighting an adaptive capacity to mitigate oxidative stress through enhanced antioxidant defenses. This adaptive response likely reflects the metabolic flexibility inherent to melanomas with higher OXPHOS reliance, as these cells are better equipped to leverage mitochondrial metabolism for redox homeostasis [25,26]. In contrast, the A375 cells demonstrated a unique metabolic shift under treatment with the two compounds, marked by increased mitochondrial ATP production, an elevated NAD^+^/NADH ratio, and an increased acetyl-CoA abundance (Figure 4). This was coupled with the upregulation of one-carbon metabolism, particularly the folate and methionine cycles, suggesting a compensatory mechanism to support nucleotide biosynthesis and redox balance (Figure 5). Such metabolic reprogramming aligns with recent reports emphasizing the role of one-carbon metabolism in the ability of cancer cells to adapt to oxidative and metabolic stress [27,28].

An intriguing aspect of these results was the difference between the two types of compounds. Aloe-Emo exhibited greater potency at lower concentrations (Figure 1), particularly in reducing cell proliferation, suggesting that subtle structural differences may influence their capacity to interfere with metabolic and redox pathways. Despite this, both compounds ultimately achieved comparable growth-inhibiting effects over prolonged treatment durations. This temporal convergence may reflect a shared ability to impose cumulative metabolic stress that melanoma cells cannot sustainably adapt to. Such findings are consistent with the literature that indicates that sustained redox disruption, rather than acute stress, is critical for effective therapeutic targeting of melanoma [29].

Our results also shed light on the intricate relationship between redox balance and melanoma survival. Both F-Emo and Aloe-Emo induced mitochondrial ROS accumulation and disrupted glutathione homeostasis, yet the extent of these effects varied. The A375 cells, reliant on glycolysis and alternative metabolic pathways such as one-carbon metabolism to support nucleotide biosynthesis under oxidative stress conditions, experienced more pronounced glutathione imbalances than COLO 800 and COLO 794. This disparity underscores the potential for metabolic dependencies to shape cellular susceptibility to oxidative stress, a concept echoed in studies linking glutamine metabolism and antioxidant defenses to melanoma resistance mechanisms [30].

Overall, our findings highlight the diverse metabolic strategies employed by melanoma cells and their distinct vulnerabilities to anthraquinone-based treatment. The observed metabolic plasticity underscores the adaptability of melanoma cells in response to therapeutic stress, emphasizing the importance of targeting multiple metabolic pathways to enhance treatment efficacy. Future studies should further explore the mechanistic basis of these metabolic adaptations and assess the potential of combining anthraquinones with other metabolic inhibitors to enhance therapeutic outcomes.

## 5. Conclusions

This study provides novel insights into the metabolic heterogeneity of melanoma cells and their responses to emodin and aloe-emodin treatment. By targeting key metabolic pathways, including glycolysis, oxidative phosphorylation, and redox homeostasis, these compounds disrupt essential survival mechanisms in melanoma cells. Notably, aloe-emodin exhibited greater potency at lower concentrations, suggesting potential structural advantages in modulating metabolic and redox pathways. The differential responses observed among the cell lines highlight the need for personalized therapeutic approaches that consider the unique metabolic dependencies of melanoma tumors. These findings contribute to the growing recognition of metabolic reprogramming as a critical target in cancer therapy and open new avenues for future investigations into combination strategies to maximize therapeutic efficacy.

## Data Availability

Requests can be made to the corresponding author for access to the data or original analysis files. The data are not publicly available as they are part of an ongoing study and will be shared upon study completion.

## Supplementary Materials

The following supporting information can be downloaded at: https://www.mdpi.com/article/10.3390/nu17071113/s1, Figure S1: (A) Representative images of COLO 800 (left panel), COLO 794 (central panel), and A375 (right panel) under the microscope. (B) Representative images of Aloe vera leaves (upper panel) and Rhamnus frangula bark (lower panel) and chemical structure of Aloe-Emo and F-Emo. (C) Autophagy levels assessed by CYTO-ID^®^ Autophagy detection kit in all three melanoma cell lines under normal conditions or under Aloe-Emo or F-Emo treatment. (D) Hierarchical clustering heatmap derived from one-way ANOVA of LC-MS metabolomics data of all three melanoma cell lines under normal conditions or after F-Emo or Aloe-Emo exposure. E) Representative images of the Western blot analysis of COLO 800, COLO 784, and A375 (normal or treated) for p-AMPKα (T172), t-AMPK, and β-actin (left panel) and their relative densitometry analyses (right panel); Figure S2: (A,B,C) Hierarchical clustering heatmaps derived from one-way ANOVA of LC-MS metabolomics data of COLO 800 (A), COLO 794 (B), and A375 (C) under normal conditions or after F-Emo or Aloe-Emo exposure; Table S1: Intracellular flux values calculated for COLO 800. Table S2: Intracellular flux values calculated for A375.

## Abbreviations

The following abbreviations are used in this manuscript:

3PG	3-phospho glycerate
5,10-MTHFD	5,10-methyltetrahydrofolate
5,10-MeTHFD	5,10-methyltetrahydrofuran
6-PG	6-phosphogluconate
6-PGDL	6-phosphonoglucono-D-lactone
AC	acetic acid
AcCoA	acetyl-CoA
ADE	adenine
ADO	2-aminoethanethiol dioxygenase
AKG	α-ketoglutarate
ALA	alanine
AMPK	AMP-activated protein kinase
ANOVA	Analysis of Variance
ARG	arginine
ASN	asparagine
ASP	aspartate
ATCC	American Type Culture Collection
ATP	adenosine triphosphate
BSA	bovine serum albumin
CDP	Cytidine diphosphate
CIS-ACO	cis-aconitic acid
CIT	citrate
CO_2_	carbon dioxide
CYS	cysteine
CYSTA	cystathionine
CP	carbamoyl phosphate
CTR	control
DCFDA	dichloro-dihydro-fluoresceine-diacetate
DHAP	dihydroxyacetone phosphate
GAP	glyceraldehyde phosphate
DMEM	Dulbecco’s modified Eagle’s medium
E4P	erythrose 4-phosphate
EAA	essential amino acid
EMU	elementary metabolite unit
F1,6BP	fructose 1,6-bisphosphate
FBS	fetal bovine serum
FCCP	carbonyl cyanide p-(trifluoromethoxy)phenylhydrazone
FOL	folate
FUM	fumarate
G3P	glyceraldehyde 3-phosphate
GLC	glucose
GLC.x	extracellular glucose
GLC-6P	glucose-6-phosphate
GLN	glutamine
GLN.x	extracellular glutamine
GLU	L-glutamate
GLU.x	extracellular L-glutamate
GLU 5P	glutamate-5phosphate
GLY	glycine
GLYC-3P	glycerol 3-phosphate
GMP	guanosine monophosphate
GSH	glutathione
GSSG	glutathione disulfide
GUA	Guanine
HCY	homocysteine
HTAU	hypotaurine
IMP	inosine monophosphate
ISOCT	isocitrate
LAC	lactate
LAC.x	extracellular lactate
LB	lower bound
LC-MS	liquid chromatography–mass spectrometry
LDH	lactate dehydrogenase
LEU	leucine
LYS	lysine
MAL	malate
MAPK	mitogen-activated protein kinase
MET	methionine
MFA	metabolic flux analysis
NAD	nicotinamide adenine dinucleotide
NEAA	non-essential amino acids
NF1	neurofibromin 1
OAA	oxaloacetate
OCR	oxygen consumption rate
OD	optical density
ORA	Over Representation Analysis
ORN	ornithine
OXPHOS	oxidative phosphorylation
P5C	pyrroline-5-carboxylate
PDH	pyruvate dehydrogenase
PFP	pentafluorophenyl
PHE	phenylalanine
PPP	pentose phosphate pathway
PRO	proline
PRPP	phosphoribosyl pyrophosphate
PYR	pyruvate
RIPA	Radio-Immunoprecipitation Assay
ROS	reactive oxygen species
RPMI	Roswell Park Memorial Institute
RU-5P	ribulose-5-phosphate
SAH	S-adenosyl homocysteine
SAM	S-adenosyl methionine
SED-7P	sedoheptulose 7-phosphate
SER	serine
SMPDB	Small Molecule Pathway Database
STDERR	standard error
SUCC	succinate
TAU	taurine
TBS	Tris-Buffered Saline
TCA	tricarboxylic acid
THR	threonine
TRP	tryptophan
TYR	tyrosine
UAC	uric acid
UB	upper bound
UDP-GLC	uridine diphosphate glucose
UHPLC-QTOF	ultra-high-performance liquid chromatography–quadrupole time-of-flight
VAL	valine
XYL-5P	xylulose 5-phosphate
