# Supplementary figures and images for: Emodin and Aloe-Emodin Reduce Cell Growth and Disrupt Metabolic Plasticity in Human Melanoma Cells
Source: Nutrients. 2025 Mar 22;17(7):1113. doi: 10.3390/nu17071113 (PMC11990439; doi:10.3390/nu17071113)

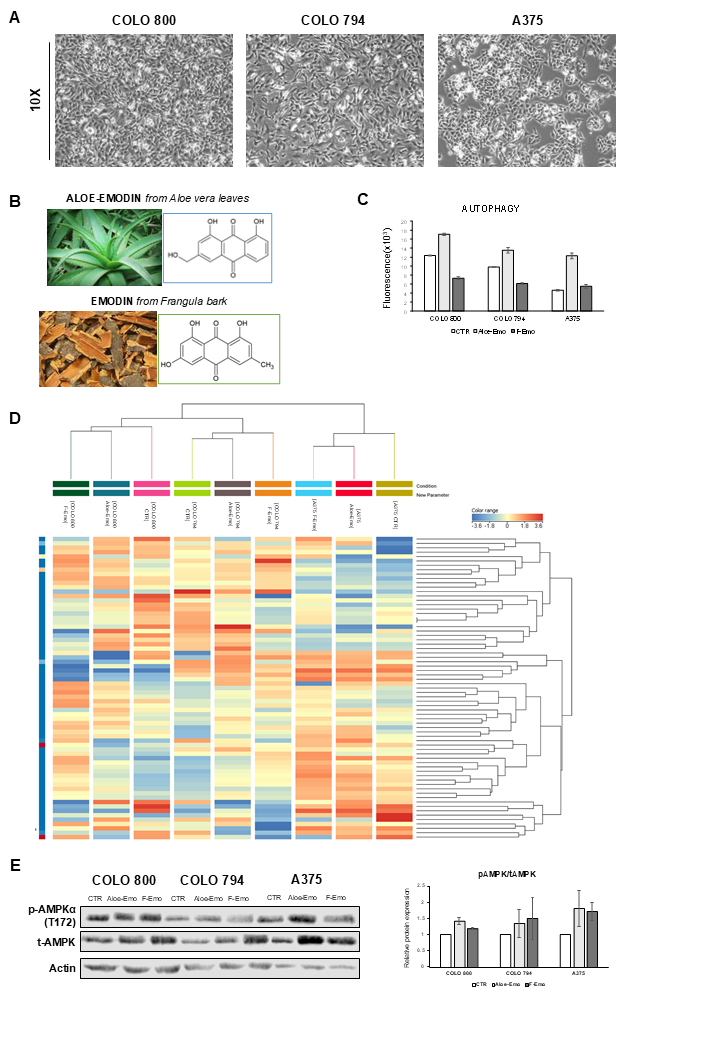

Supplement: Supplementary file 1 [file nutrients-17-01113-s001.zip › Figure S1.TIF]

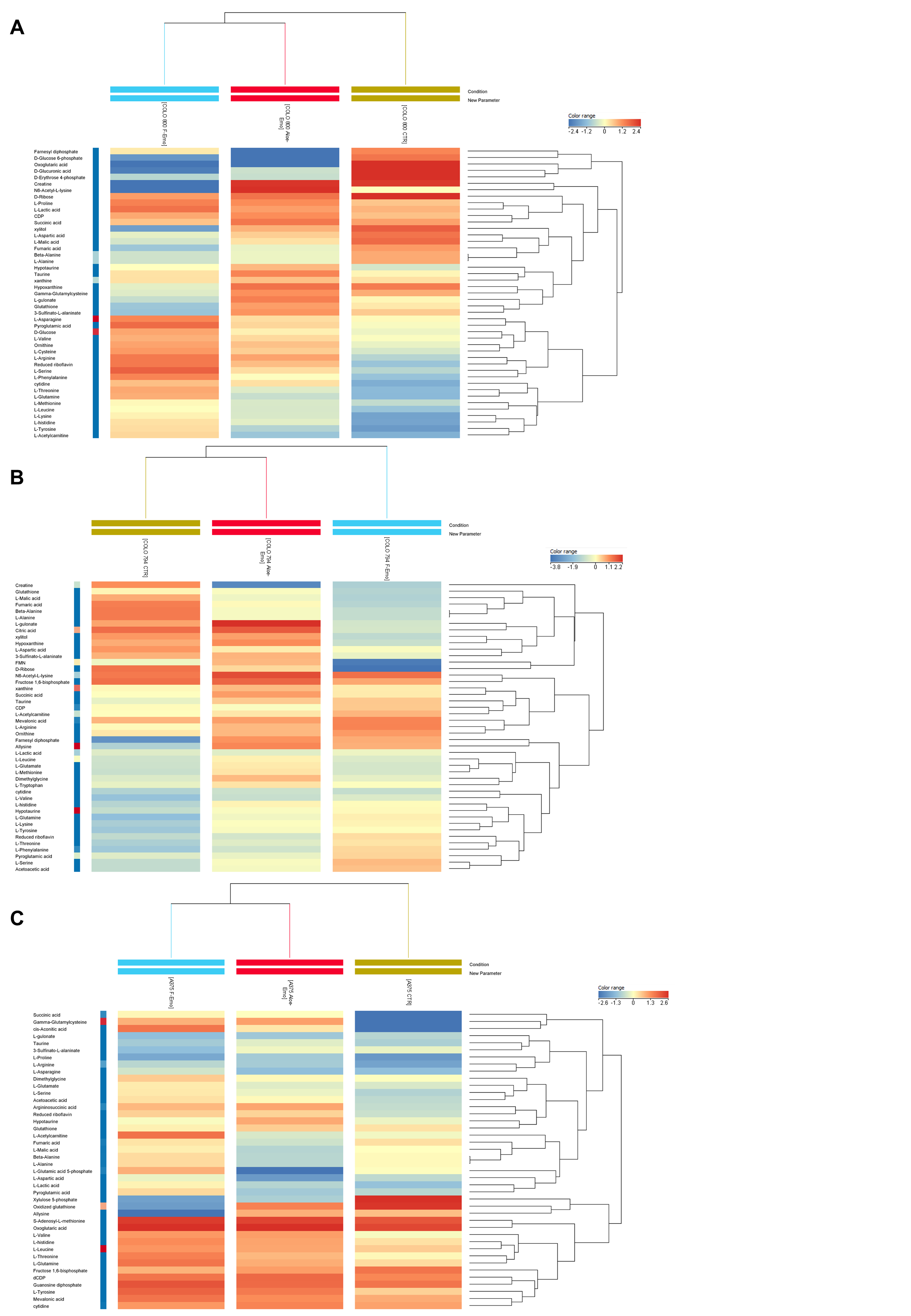

Supplement: Supplementary file 1 [file nutrients-17-01113-s001.zip › Figure S2.TIF]
